# Causal Inference in Studies with Functional Unmasking: Psychedelics and Beyond

**DOI:** 10.64898/2025.12.05.25341713

**Published:** 2025-12-08

**Authors:** Gabriel Loewinger, Mats J. Stensrud, Sandeep M. Nayak, David Yaden, Alexander W. Levis

**Affiliations:** 1 Machine Learning Core, National Institute of Mental Health; 2 Institute of Mathematics, École Polytechnique Fédérale de Lausanne; 3 Center for Psychedelic and Consciousness Research, Johns Hopkins University School of Medicine; 4 Center for Causal Inference, Department of Biostatistics, Epidemiology and Informatics, University of Pennsylvania

**Keywords:** psychedelics, psychiatry, causal inference, randomized controlled trials, semi-parametric mediation analysis

## Abstract

In clinical trials for mental health treatments, functional unmasking (unblinding) is a widespread challenge wherein participants become aware of their assigned treatment. Unmasking is especially concerning with psychedelics, due to the near unmistakable acute effects (the “trip”), resulting in uncertainty about whether outcomes following treatment reflect true therapeutic properties of the interventions, or placebo-like effects. We present a counterfactual conceptualization of unmasking that 1) formalizes the shortcomings of many existing statistical and experimental design solutions (e.g., dose-response, active controls), and 2) demonstrates how modern causal inference approaches can be applied to isolate effects devoid of this “contamination.” Our results reveal feedback mechanisms between perceived therapeutic benefits and expectancies that can render traditional methods prone to obscuring or exaggerating therapeutic benefits. Our proposal motivates trial designs and statistical methods that can be implemented to mitigate the impacts of functional unmasking.

## Introduction

Participant functional unmasking (or unblinding) frequently arises in mental health research, where acute effects of a treatment often allow participants to accurately guess their treatment assignment. This, in turn, can lead to differing “hopes” for improvement across treatment arms, possibly affecting study conclusions. At the heart of this discussion lie questions about causal effects: 1) does the compound itself *cause* therapeutic benefits, or 2) are functional unmasking and resulting discrepant hopes across treatment arms responsible for treatment effects. On the one hand, randomization ensures that average between-arm differences in the outcome are caused by treatment; the hopes, themselves, can be cast as part of the therapeutic mechanism or treatment effect. However, if hopes, or *expectancies*, strongly influence treatment effects, some may be concerned that therapeutic benefits may not be durable, or that study results may generalize poorly if these expectancies change across time or populations. We argue that introducing specific formal summaries of treatment effects (*causal estimands*) helps clarify and address the issue of unmasking.

**Figure 1:**
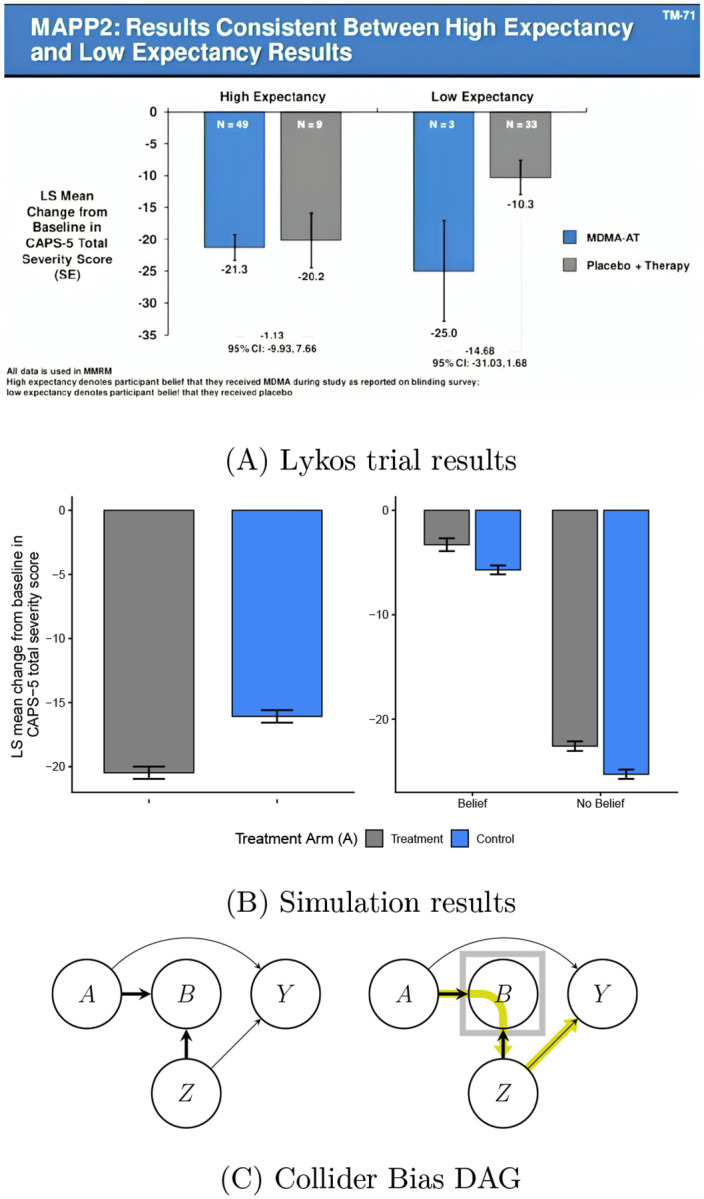
Bias from analyzing results stratified on post-treatment variables. (A) Slide presented by a statistical consultant to Lykos at the FDA Advisory meeting hosted on June 4, 2024. (Source: https://psychedelicalpha.com/news/live-coverage-fda-advisory-committee-reviews-mdma-assisted-therapy-for-ptsd). This figure was also reproduced in Colloca and Fava (2024) who note that “Presented is LS [Least Squares] mean change in the clinician-administered PTSD scale for DSM-5 (CAPS-5) total severity score.” (B) Simulation results to illustrate bias from stratifying on post-treatment variables (e.g., belief). Error bars indicate 95% confidence intervals. [Left] We compare the change from baseline PTSD scores (as shown in (A)) across treatment arms. There is a strong PTSD reduction from treatment, showing that the treatment was simulated to be beneficial; concretely, the controlled direct effect (as introduced later in the paper) of treatment, fixing belief, was negative (beneficial) for all subjects. [Right] The within-belief level differences, which wrongly makes it appear as if the treatment is instead harmful (i.e., the treatment effect sign is opposite to that of the left panel). The discrepancy between the two figures illustrates how stratifying on post-treatment belief can lead to such strong bias that the analysis suggests the opposite conclusion: the data are simulated with a large beneficial effect of treatment (negative) but the stratified analysis makes it wrongly appear as if the treatment effect is harmful (positive). This is an example of collider bias. We detail this simulation setting in Appendix E.4.2. (C) Directed Acyclic Graphs (DAGs) to illustrate collider bias in comparison of treatment effect estimates across stratum of post-treatment beliefs/expectancy. We use the notation A→B to express that treatment, A, is a cause of post-treatment belief, B. In this DAG, we denote Z to be common causes of belief B and the outcome variable Y (e.g., pre-treatment expectancies). The DAG on the left illustrates how an analysis that omits post-treatment belief B (e.g., comparing the mean outcome across treatment arms) is valid: post-treatment variables B are *colliders* on the A→B←Z path (represented by the thick arrows). This *blocks* confounders, Z, of the B→Y pathway, from exerting causal effects mediated through the A→Y pathway (i.e., the path from A→B←Z→Y is blocked). The DAG on the right illustrates why stratifying treatment effect estimates on B (or including B in a regression without formal causal estimation methods) invalidates this analysis: stratification on B (represented by the box) *opens* the pathway from A→B←Z→Y, thereby introducing bias from Z and B. Thus conditioning on B in this manner could lead to a beneficial treatment appear (incorrectly) harmful.

Whether functional unmasking invalidates causal inferences from randomized clinical trials (RCTs) is a debate that is especially active in psychedelic research due partly to the FDA’s 2024 rejection of the Lykos pharmaceutical application for midoamfetamine (MDMA) as a treatment of PTSD. The FDA advisory committee’s concerns over study bias from expectancy effects and functional unmasking (Colloca and Fava, 2024) have led to a slew of publications (Colloca and Fava, 2024; Rosenbaum, 2024; Nayak and Zahid, 2025; Schenberg, 2025; Szigeti et al., 2024a) and dedicated meetings (Cheung et al., 2025; Reagan-Udall Foundation for the FDA, 2024). To illustrate, a statistical consultant to Lykos presented an analysis, displayed in Figure 1A, at the FDA meeting showing that treatment effects (i.e., differences in follow-up PTSD levels between treatment arms) varied substantially depending on whether patients believed they had received MDMA. Similar analyses have been applied in other psychedelic RCTs to compare treatment effects across levels of post-treatment beliefs or expectancies (Bershad et al., 2019; van Elk et al., 2022; Lii et al., 2023a; Cavanna et al., 2022). Intuitively, if these analyses reveal larger effects among those who believe they received active treatment, it is tempting to conclude that placebo effects are inflating estimates of the therapeutic benefits. We appreciate that these analyses are often exploratory, but we caution that causal theory reveals that such approaches can be highly misleading due to so-called *collider bias*. In fact, we illustrate in Figures 1B–C and Appendix E.4.2 that conditioning on post-treatment variables in this manner (i.e., stratifying analyses on these variables or including them as covariates in regression models without formal causal estimation tools) can obscure treatment effects so strongly that highly beneficial treatments wrongfully appear as if they were harmful. This example, in addition to other common pitfalls that we have observed in the literature, highlight that formal causal theory is needed to correctly estimate the desired causal effects in studies with functional unmasking.

Carefully applying causal tools can allow recovery of treatment effect estimates in psychedelic trials comparable to those that would be obtained in a successfully masked study. In other domains, applications of tailored causal methods to observational data have yielded treatment effect estimates comparable to those from subsequent RCTs. Causal inference methods have proved critical for demonstrating treatment efficacy in both high-profile RCTs and observational studies of, for example, HIV (Robins, 1987, 1986), cancer (Dickerman et al., 2019), hormone therapy in women (Hernán et al., 2008), and COVID-19 (Dickerman et al., 2022). Together, the widespread applications and successes of causal inference have resulted in the awarding of the Turing award in 2011 (Pearl, 2011), Nobel prize in 2021 (Organization, 2021), and Rousseeuw Prize for Statistics in 2022 (King Baudouin Foundation, 2022b,a). These successes were enabled in part by modern causal estimation methods based on semiparametric theory that can incorporate powerful machine learning algorithms. These tools have not, however, been widely adopted to combat methodological challenges in mental health research.

We aim to apply *counterfactual* and *graphical* causal theory to 1) argue that statistical approaches and RCT designs (e.g., dose-response, active controls/comparators, treatment under anesthesia) applied in the psychedelic literature will not alone resolve the unmasking challenge, 2) propose modern causal inference methods to account for unmasking in existing RCT designs, and 3) suggest new designs that can mitigate and/or quantify the effects of unmasking. As a solution to a challenging instance of functional unmasking, our work can be adapted to studies of treatments beyond psychedelics.

## Expectancy: Nuisance or Mechanism?

To introduce our proposal, we first define functional (un)masking in formal causal language, illustrated by an example hypothetical RCT studying treatment for depression. A sample of participants are randomized at baseline to receive a single dose of psilocybin, or methylphenidate as an active comparator/control. Following standard causal inference notation (Hernan and Robins, 2024), we denote the treatment (psilocybin) arm as A=1 and the control (methylphenidate) arm as A=0. To focus on the main arguments, we assume no dropout and full adherence to the assigned treatment. We measure baseline (pre-treatment) covariates, X, like “hype” (pre-treatment hopes), age, and sex. Six months after treatment, we measure follow-up depression levels, Y, and compare the averages of this outcome variable across arms to estimate the *average treatment effect* (ATE). Shortly after treatment, we also measure: 1) *beliefs*, B, indicating which treatment a participant thinks they received; 2) post-treatment *expectancies*, E, indicating whether they think their depression Y will improve/worsen (see Appendix A for a comparison of these definitions with previous work); as well as 3) a collection of variables (denoted by Z) that affect post-treatment expectancy and the outcome. The vector Z might include, for example, the experience of the trip. To avoid confusion, we use *hype* to refer to pre-treatment hopes, and *expectancy* (E) to refer to post-treatment hopes. To introduce counterfactual causal reasoning, we define the *counterfactual* (or *potential outcome*) Y(a) which denotes the outcome (depression levels) that *would be* observed if, potentially counter-to-fact, an individual received treatment level a. We use uppercase A to denote the actual observed treatment value, and lowercase a for the intervention value that we set the treatment to. This frames causal inference in terms of a hypothetical setting where we could observe follow-up depression levels of a given participant in two worlds: one in which they received psilocybin and one in which they received control. Although we cannot observe data from both worlds for the same individual, we can use real data to estimate average outcomes under each treatment condition. For example, in our psilocybin RCT, we can estimate the average of each counterfactual as the mean outcome in the respective treatment arm. More generally, our strategy for targeting more complex causal questions proceeds through (i) articulation of the research question of interest, formalized as a causal estimand, and (ii) delineating when and how to estimate it using data from new or existing experiments.

We use this framework to argue that resolving the functional unmasking debate boils down to carefully accounting for (post-treatment) expectancy, E, statistically or experimentally. The expectancy/belief distinction is critical because it clarifies why treatment effect estimates can be “contaminated” even when masking is successful. In fact, to those skeptical of psychedelic’s therapeutic benefits, randomized open-label (unmasked) studies without expectancy effects may be less worrisome than successfully masked studies where the treatment/control cause differing expectancy levels. For example, consider a randomized open-label study comparing participants’ absorption levels of two Vitamin E oral supplement formulations that have equivalent perceptible effects (e.g., side effects). If neither formulation is expected to be superior, knowledge of which one a participant has received is unlikely to systematically favor one treatment. We call this study *belief unblinded*, as participants can determine which arm they were assigned to, and *expectancy blinded*, as each participant in this study is assumed to have the same expectancy levels regardless of treatment assignment. On the other hand, *belief blinded* – *expectancy unblinded* RCTs appear more concerning. In such studies, treatments cause differences in expectancy levels across arms even though participants cannot determine which treatment they received. For instance, consider the (potentially unrealistic) depression treatment study where salvia is used as an active control/comparator for smoke-able DMT among a psychedelic-naive sample. Suppose salvia’s psychoactive effects lead to belief blinding, but its dysphoric properties lead to negative expectancies about how treatment will influence depression levels (i.e., expectancy unblinding). Then, even if neither DMT nor salvia possessed lasting therapeutic benefits, the outcomes of this hypothetical RCT may suggest DMT is beneficial only because salvia led to more negative expectancy levels than DMT.

**Figure 2:**
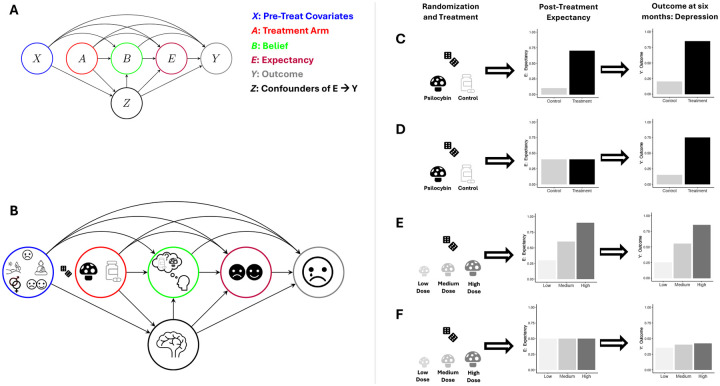
Causal conceptualization of psychedelic trials in the presence of functional unmasking. (A) Causal directed acyclic graph (DAG) (Hernan and Robins, 2024) the hypothetical psilocybin RCT with variables: X – baseline (pre-treatment) covariates; A – treatment (A=1) control (A=0) arm; B – participant belief that they received treatment (B=1) or control (B=0); Y – outcome variable (e.g., depression); E – expectancy about how they think their outcome will change from treatment; Z – post-treatment confounders of E→Y path (i.e., post-treatment common causes of both E and Y). The direction of B→E instead of E→B may be switched depending on the study design and source of unmasking. This DAG allows one to define belief-, expectancy-, and complete-blinding in terms of causal pathways (see Appendix Table 1). (B) The same DAG as in A but with graphical illustration of example variables to include. X contains pre-treatment depression, set and setting, biological sex, and hype (pre-treatment hopes/attitudes). Z contains the acute effects of the trip (represented by the brain). A has a dice icon next to its node to indicate no arrows point into it because of randomization (i.e., there are no causes of A). Post-treatment expectancy E is indicated by smiling/frowning face. Y is post-treatment depression. (C) Sequence of 1) randomization/treatment, 2) resulting expectancy changes, and 3) the outcome. This sequence illustrates how active (A=1) and control (A=0) treatments often result in changes in both expectancy and the outcome leading to the possibility that expectancy *mediates* treatment effects. (D) Illustration of the idealized setting where even when (post-treatment) expectancy is experimentally “set” to be the same value for a given individual regardless of treatment/control arm assignment, the treatment effect (differences in the outcome across arms) remains pronounced. This illustrates a setting where expectancy does *not* mediate treatment effects substantially. However, if expectancy *does* mediate these effects, then other approaches are needed to account for expectancy effects. For example, (E)-(F) illustrates why dose-response studies do not necessarily resolve the functional unmasking problem: expectancy may itself exhibit a dose-response relationship with the treatment. Thus, the dose-response effect in the outcome may be mediated by this expectancy dose-response profile. (F) shows what might be observed in practice (without experimentally controlling expectancy). (G) shows what would happen in a psychedelic dose-response study if therapeutic effects are in fact entirely mediated through expectancy: we experimentally “set” expectancy to be the same across treatment doses (within an individual) and the treatment effects on *Y* disappear because they were almost entirely mediated through the dose-response expectancy relationship.

To analyze these ideas more precisely, we use the variable definitions from our hypothetical psilocybin RCT and encode their relation in a directed acyclic graph (DAG) (Hernan and Robins, 2024) in Figure 2A. In the DAG, the notation A→B expresses that treatment, A, is a cause of belief, B. The DAG shows how all past variables are potential causes of all future variables, except A: treatment has no causes because of randomization (no arrows point to A). Thus, baseline variables X, like hype, might modify the effects of A on Y but do not *confound* the A→Y pathway (even in the presence of functional unmasking) because X is not a *common cause* of these variables.

These DAG paths distill complex mechanisms without discarding critical information. For example, the A→Y or A→B pathways may be mediated by side-effects, which relate to benign/malicious unmasking (Howick, 2011), as discussed below. Similarly, the effect of expectancy on depression (E→Y path) may be mediated by behavior change (e.g., diet). There may also exist biological/psychological mechanisms (i.e., causal pathways) that are not mediated through E (e.g., represented by the direct A→Y path), but are critical to psilocybin’s effectiveness. Thus, although simplified, a more extensive DAG that includes these intermediate mechanisms does not, in most cases, change the causal structure necessary to identify how to mitigate the impacts of functional unmasking.

Whether treatment effects are or are not mediated through E may seem arbitrary to someone who views expectancy effects as a key mechanism of psychedelics. We argue, however, through the formal approach we propose, that understanding the magnitude/durability of the causal effects of treatment and (post-treatment) expectancy together can directly probe the concerns of critics (e.g., Muthukumaraswamy et al. (2021); Butler et al. (2022)) that unmasking is unfairly driving the large effect sizes reported. For instance, a skeptic that views expectancy as a *nuisance* might only be convinced of psychedelics’ therapeutic potential if treatment effects are shown to be large in a study where E was effectively fixed to be high (“hopeful”) or low (“not hopeful”). Conversely, those who conceptualize E as a legitimate *mechanistic* component of the treatment, may seek to demonstrate that the independent therapeutic benefits of expectancy are durable enough to be considered a reliable therapeutic effect. Thus, for both the nuisance and mechanistic camps, estimating the joint effects of treatment and expectancy lies at the foundation of the unmasking debate.

## A Counterfactual Description of Functional Unmasking

Our counterfactual framing clarifies *why* accounting for expectancy mitigates the downsides of functional unmasking, *what* quantitative summaries of treatment effects (causal estimands) isolate these unwanted expectancy effects, and *how* to estimate them in RCTs. We start with the *why*. Our approach reveals that expectancy is a causal mediation problem, not a confounding problem: since unmasking is a post-treatment phenomenon, E and B are intermediate variables between A and Y, not common causes (confounders) of A and Y (see Figure 2 for intuition). This conflicts with what we believe is a common misconception that unmasking *leads to* confounding. For instance, Muthukumaraswamy et al. (2021) state that “[p]sychedelic RCTs are likely confounded by de-blinding” and if the RCTs “cannot be adequately masked…[they] will always be confounded.” We argue that “confounding” of the treatment–outcome relationship will not occur in RCTs from unmasking because randomization ensures there exists no common causes of the treatment and outcome (i.e., no confounding). Thus, RCTs enable valid estimation of the ATE even under unmasking. That ATE may, however, be partially *mediated* through unwanted (post-treatment) expectancy effects. This emphasizes why accounting for hype (pre-treatment expectations) may not, on its own, fully address the unmasking challenge. For example, Aday et al. (2022) write that “without effective condition masking, it is virtually impossible to maintain the independence of the […] [treatment], as it is confounded by participant [pre-treatment] expectations.” But A is randomized so hype cannot *confound* the A→Y pathway. In fact, hype will be balanced on average across arms. Thus, statistically/experimentally controlling for hype will not alone resolve the unmasking challenge. Hype *is* likely critical to incorporate into analyses, but as a confounder of the E→Y relationship. For example, unmasking may “unleash”/“unlock” pre-treatment hopes/attitudes to strongly influence (post-treatment) expectancy and depression.

Second, by conceptualizing treatment effects as mediated through expectancy, our framing provides formal statistical methods and RCT designs to isolate study effects of treatment that control expectancy (the *what*). Specifically, the *controlled direct effect* (CDE) causal estimand isolates this effect. We discuss the formal definition below, but conceptually, the CDE quantifies the average between-arm difference in Y, in a hypothetical study where we experimentally *set* (post-treatment) expectancy levels to the same value across arms. Formally defining the causal effects that we target is critical: while others have appealed to DAGs to describe unmasking in psychedelic RCTs, we believe that these proposals have drawbacks. In fact, as discussed below, some of these methodological proposals (e.g., Szigeti and Heifets (2024)) can result in estimates that are so misleading, they can make beneficial treatments appear harmful, or vice-versa (see Appendix E.4.1 for a detailed derivation).

Third, the DAG clarifies that we can estimate the CDE if the correct variables are measured, or if certain RCT designs are implemented (the *how*). Specifically, in RCTs that only randomize A, one must measure and adjust for all common causes (X and Z) of expectancy E and depression levels Y. We discuss what these variables might be below (e.g., X should include hype). Unlike past proposals in the psychedelic literature, we propose application of formal and modern causal inference methodology. Importantly, estimation differs from the intuitive, but invalid, strategy of *conditioning* or *stratifying* on a measurement of expectancy. The DAG also motivates that one can instead experimentally manipulate E with specific types of RCT designs (discussed below). Thus these statistical and experimental strategies allow one to isolate treatment effects devoid of unwanted expectancy “contamination.”

## Classical RCTs do not always target the right causal effect

Before discussing estimands to account for unmasking, we describe drawbacks of the ATE. Because expectancy-mediated effects are often unwanted, the ATE does not always summarize the effects of interest. Conceptually, this is because the average *total effect* of A→Y may be composed of a *direct effect* of A→Y and *indirect effect* mediated through E:A→E→Y. Thus the indirect effects mediated through expectancy may exaggerate or obscure the direct (biological/psychological) effects of psychedelics that are not expectancy-mediated. In fact, treatment can induce expectancy feedback mechanisms that further complicate the interpretation of total effects.

**Figure 3:**
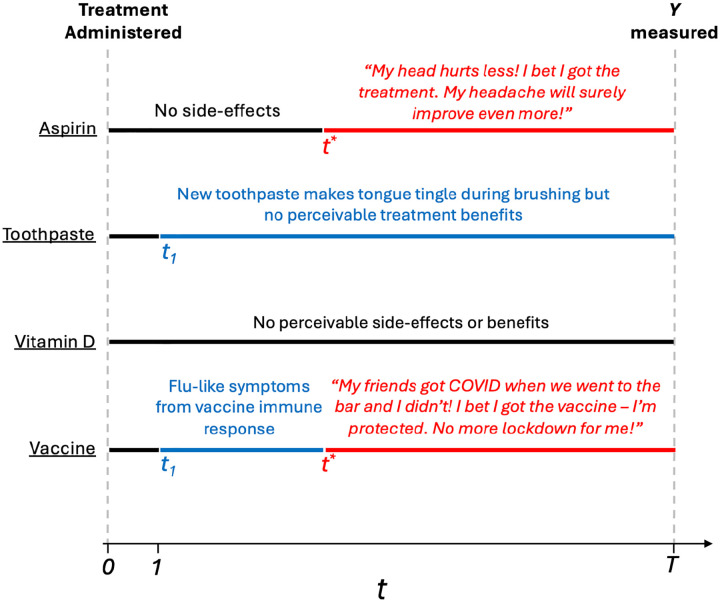
Four example RCTs with and without side-effects and/or efficacy-expectancy feedback. In all examples, the (horizontal) black line indicates the time-interval between treatment administration (t=0) and the first perceivable effects (either $t_{1}$ or $t^{*}$). Here, $t_{1}$ is the time when perceivable side-effects occur. $t^{*}$ is the time when perceivable therapeutic benefits occur. T is study follow-up. The blue line indicates intervals from $t_{1}$ until either $t^{*}$ or T. The red line indicates time-windows between $t^{*}$ and T. The lack of a red line means the treatment has no perceiveable therapeutic benefits. The lack of a blue line means the treatment has no perceiveable side-effects. Aspirin We run a double blind RCT to study the efficacy of aspirin, relative to an inactive control, to treat headaches. *Aspirin* is effectively masked and has no perceivable effects until $t^{*}$, the timepoint, aspirin (A=1) arm participants begin to feel improvements in their headache. When aspirin arm participants begin experiencing a “real” treatment-induced reduction in headaches, a “snow-balling” effect occurs: the therapeutic benefit leads to greater expectancy. These patients think “my headache is improving, I bet I got the active treatment, and my headache will improve even more.” This greater expectancy further improves the perceived efficacy of the treatment, which again feeds back leading to even greater expectancy levels. This describes *efficacy-expectancy feedback*. The Toothpaste RCT compares two toothpastes for gum health at six month follow-up. The A=1 arm toothpaste has side-effects (e.g. the tongue “tingles”) (starting at $t_{1}=1$) that the comparator toothpaste (A=0) does not. However, treatment benefits to gum health are not perceptible during the study and so there is no efficacy-expectancy feedback. The Vitamin D Supplement RCT compares two formulations. Neither treatment have any perceivable effects. The *COVID-19 Vaccine* induces flu-like symptoms the day after administration because of the acute immune response. Six weeks after administration, treatment arm (A=1) participants notice that they did not develop COVID symptoms when exposed, but their unvaccinated friends got infected. As a result of their perceived protection, participants begin taking more risks (e.g. going to restaurants more often), inducing a form of efficacy-expectancy feedback. This increased exposure to infected individuals lead to greater infection, masking the vaccine-induced immunity. Appendix Table 2 describes the blue and red time-intervals in terms of a 2 × 2 table of side-effect timing vs. efficacy-expectancy feedback.

We illustrate this concept via several RCTs in Figure 3. This phenomenon, which we term *efficacy-expectancy feedback*, describes how perceived post-treatment efficacy of the treatment affects (post-treatment) expectancy, in turn impacting the outcome itself. In some cases, this creates a “snow-balling” whereby perceived treatment benefits cause greater expectancy, feeding back to a greater perception of treatment efficacy (see the *Aspirin* example in Figure 3). Efficacy–expectancy feedback (formalized in Appendix D.1) can be cast as a special case of treatment-confounder feedback, a well-known causal inference concept (Robins, 1986; Hernan and Robins, 2024), whereby feedback mechanisms can also obscure treatment effects (Levis et al., 2024).

**Figure 4:**
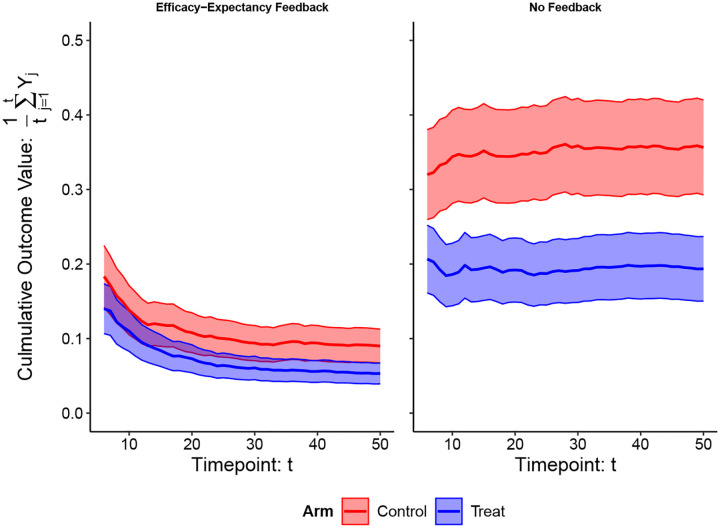
Efficacy–Expectancy Feedback Example Vaccine RCT simulation example. [Left] Simulation results *with* efficacy-expectancy feedback: participants reduce their sexual risk behavior if they become infected. The outcome $Y_{j}=1$ if a participant became infected at timepoint j and $Y_{j}=0$ if they did not become infected. We show the average culmulative number of infections on the y-axis and timepoint on the x-axis. [Right] Simulation results from an identical simulation but *without* efficacy-expectancy feedback: participant infection throughout the study does *not* alter their risk behavior. When participants have the same risk behavior in both arms: the vaccine reduces the risk of infection for each exposure by 50%. This simulation illustrates a vaccine RCT for the sexually-transmitted infection Chlamydia among freshman college students on a campus with an outbreak. All freshman students are randomized to receive either the vaccine (A=1) or a control (A=0) shot before the first semester. There are no side effects and successful belief-blinding is achieved throughout. Participant infection status is tested weekly, and infected participants are given an antibiotic to treat the infection. For simplicity, we imagine the antibiotic works instantaneously, and does not prevent future infections. At the end of the study, the average number of infections each participant experienced is compared across arms. Efficacy-expectancy feedback can obscure strong treatment effects: testing positive for Chlamydia scares students into reducing their tendency to have unprotected sex. The tests therefore provide “feedback” in a manner that can alter behavior, even if participants are unsure of which treatment arm they are in. Since the data is simulated under a scenario where the vaccine is effective, A=1 students become infected with a lower probability for each exposure, and so over time, they exhibit riskier behavior than controls on average (i.e., vaccine participants are “scared-off” from future unprotected sex less than controls). We provide simulation details in Appendix D.2.

The hypothetical RCTs in Figure 3 also illustrate important time-windows and differentiate between 1) unmasking from perceivable side-effects versus from therapeutic benefits, and 2) expectancies before/after perceived therapeutic benefits (“efficacy”). We argue that this pre-efficacy/post-efficacy distinction is preferable to the benign/malicious unmasking dichotomy, defined as unmasking from perceived efficacy, or side-effects, respectively (Howick, 2011). Indeed, unmasking due to perceived treatment efficacy is often considered “benign”, but it can also exaggerate/shroud therapeutic benefits. In Appendix D, we extend the DAG in Figure 2A with longitudinal measurements of E and B to formalize these critical time-windows, and distinct types of post-treatment expectancy effects. In Figure 4, we illustrate an example of efficacy-expectancy feedback in a mathematical simulation of an RCT in which the immunity from a vaccine contributes to higher risk-taking behavior than control arm participants, even when participants are effectively belief-blinded. The higher risk-taking behavior, an example of expectancy unblinding, counteracts the vaccine-induced protection, ultimately obscuring the protectiveness of the vaccine when quantified with an ATE. However, a CDE estimate reveals the underlying vaccine effectiveness: vaccinated participants would exhibit far lower incidence than controls if we had intervened (e.g., with education) to ensure subjects in both arms had identical risk behavior (i.e., expectancy levels).

This concept and the above example are relevant to psychedelic RCTs. First, belief-blinding is not sufficient to prevent expectancy feedback. Even when between-arm differences in expectancy levels arise solely from treatment efficacy (e.g., higher risk behavior resulting from vaccine protectiveness), treatment effects can still be obscured or exaggerated. Thus, even if functional unmasking in psychedelic RCTs resulted solely from a recognition of the therapeutic benefits from the trip (i.e., no placebo effects), controlling expectancy effects would still be desirable for both nuisance and mechanistic camps. Finally, this shows how careful causal analyses can reveal both expectancy feedback and therapeutic benefits that would otherwise be obscured if summarized with an ATE.

## Flexible Causal Analysis

Some of the major successes of causal inference in high-profile biomedical studies were enabled by causal methods to resolve feedback mechanisms (Robins, 1986; Hernan and Robins, 2024) akin to efficacy-expectancy feedback, highlighting the potential promise of our proposed strategy to use these tools to isolate the effect of expectancy. Specifically, we can formalize the unmasking challenge as a causal analysis of questions like “what is the difference in depression levels at follow-up among those treated and those not treated with psilocybin, in a world where we set expectancy levels to the same value across arms?” This describes, at an individual participant-level, what the causal effect of psilocybin levels are (on depression) for a fixed level of expectancy. The average of these individual-level differences is the *controlled direct effect* (Robins and Greenland, 1992), defined by the contrast of counterfactual means,

(1) $$E\left[Y\left(a=1,e\right)-Y\left(a=0,e\right)\right].$$

The CDE represents a contrast of effects of two joint interventions: one that sets A to active treatment (A=1) and expectancy to E=e, and another that sets A to control (A=0) and expectancy to E=e (illustrated in Figure 6A). The CDE can be conceptualized by an idealized *target trial*: a hypothetical experiment that naturally enables estimation of the causal effect that we seek to estimate by jointly intervening on the variables of interest (i.e., both the treatment and expectancy levels). For example, imagine the 2 × 2 design of (psilocybin vs. methylphenidate) × (low vs. high expectancy), where experimentally setting expectancy exactly to a low/high level was feasible with, for example, a consultation with a trusted physician that effectively convinces participants to be hopeful or neutral. With such a design, one could compare average depression levels Y across psilocybin/methylphenidate sub-arms, within the low- or high-expectancy arm. This would allow one to experimentally test for the effect of psilocybin while “controlling” for expectancy effects. This 2×2 design illustrates how a hypothetical expectancy manipulation is *theoretically* well-defined, even if perfect manipulation is not practically feasible (or ethical) at the time of the trial. Critically, the CDE can, in principle, be estimated in two ways: from trials like the 2 × 2 design above that experimentally manipulate expectancy, or from an RCT that only manipulates A (psilocybin/methylphenidate), by applying observational causal inference techniques.

To experimentally manipulate expectancy, one could deliver auxiliary sham treatments alongside the psychedelic/control. For example, one could randomize whether or not to deliver an inactive placebo like fish oil, paired with the *message* “this auxiliary drug has no effects on the psychedelic trip but may increase (decrease) the psychedelic’s anti-depressant qualities by enhancing (blocking) neurogenesis after the trip.” This has the advantage of altering expectancy, without the risk of acute harm to patients. By using a fake pill, it may also be a more efficacious expectancy manipulator. Since fish oil has putative neurogenesis effects (Wen et al., 2024), it may satisfy deception-related ethical concerns. Messages have also been proposed in vaccine RCTs to mitigate unblinding Stensrud et al. (2024); Obolski et al. (2024). If these or other existing strategies cannot perfectly experimentally set/manipulate expectancy levels, as some have suggested (Szigeti and Heifets, 2024), a contrast of average outcomes across arms for a fixed message will *not* correspond exactly to the CDE (see discussion of interpretations and statistical tools for such experiments in Appendix E.5). Nevertheless, even if expectancy levels cannot be perfectly set experimentally, assessing the joint impact of treatment and *practical* expectancy manipulations can help probe the impact of unmasking by testing the sensitivity of treatment effect estimates to interventions on expectancy.

Alternatively, the CDE can—under transparent assumptions—directly be estimated from even a standard trial (where only psilocybin/methylphenidate is randomized) using techniques from observational causal inference. Thus, we can estimate CDEs from trials that have already been conducted. A key assumption is that E is “as good as randomized” within levels of X,A, and Z. In the psilocybin example, it would likely be critical to include assessments of 1) anticipated pre-treatment hopes under both placebo and psilocybin in the vector X, and 2) immediate perceived effects of the treatment and (post-treatment) belief in the vector Z. For those that view expectancy effects as a key mechanistic component of psychedelics, one might also estimate the *treatment-fixed CDE of expectancy* (tfCDE), which quantifies the independent causal effect of expectancy within-arm. This is defined by the contrast $E[Y(a,e)-\left.Y\left(a,e^{\prime }\right)\right]$ across two expectancy levels $e,e^{\prime }$, for a fixed level of treatment a. In Appendix E, we state assumptions formally and discuss implications. Critically, the CDEs/tfCDEs cannot be estimated by stratifying analyses on expectancy/treatment levels or by including them as covariates in a regression model.

Importantly, the timing of expectancy measurements determines the interpretation of the CDE and the E→Y confounders that must be measured.

- **Pre-efficacy Effect Isolation** In some studies, the causal effect of pre-efficacy expectancies E (i.e., those occurring before onset of perceivable therapeutic benefits), can be isolated. This might be feasible if, for example, psilocybin’s anti-depressant effects were caused by neurogenesis processes that do not result in perceivable changes in depression until several days after treatment. Then expectancy levels measured a day after treatment could be conceptualized as pre-efficacy, and used to estimate a pre-efficacy CDE: the effect of the treatment under a fixed level of pre-efficacy expectancy changes. This would control for the effects of the “trip” on expectancies pre-efficacy, but not for post-efficacy expectancy changes from perceived therapeutic benefits.
- **Post-efficacy Effect Isolation** If therapeutic benefits are instead mediated through the trip, itself, then efficacy-expectancy feedback begins at trip onset ${(t}_{1}=t^{*})$ and one must therefore adjust for all common causes of post-efficacy expectancy and the outcome. In addition to pre-efficacy expectancy confounders, one would need to measure mediators of efficacy-expectancy feedback, like initial post-treatment measurements of Y (e.g., depression levels recorded after treatment but before the expectancy measurement). This formalizes how disentangling expectancy effects due to placebo effects versus perceptions of therapeutic benefits is more difficult post-efficacy.

In Appendix A.3, we discuss why we have centered our approaches for functional unmasking around CDEs/tfCDEs. For example, we describe why the well-known “Natural Direct Effect” is less relevant and unlikely to be identifiable in psychedelic studies (Robins and Greenland, 1992; Pearl, 2001). There are also related questions that correspond to estimands different from the CDE, such as *separable causal effects*. For example, suppose one sought to isolate distinct components of a psychedelic’s therapeutic effects attributable to the “the trip” versus non-psychoactive effects. This objective aligns with study designs that aim to “add the trip onto the control” (e.g., with active comparators) or “take the trip out of the drug” (e.g., co-administration of anesthesia (Lii et al., 2023b) or haloperidol (Vollenweider et al., 1998)). These designs implicitly rely on a conceptualization of the trip-related and non-trip-related effects as causally *separable* (Robins and Richardson, 2010; Robins et al., 2022; Stensrud et al., 2023, 2022), which would be justifiable if, for example, the components were generated by distinct neurobiological mechanisms.

## Statistical Mediation Analysis in Practice

Our proposed approach to expectancy-adjustment is based on modern causal inference methods that rely on fewer assumptions and have better statistical properties than classical regression-based approaches (see Appendix E.2 for a detailed explanation). This is critical as parametric approaches, like including confounders as covariates in a parametric model (e.g., linear/logistic regression), are only valid under strong, likely unrealistic assumptions. By using flexible machine learning methods as part of CDE estimation, one can capture complex interactions and non-nonlinearities without assumptions about the functional form of complex relationships between measurements of Z (e.g., trip “experience”), X (e.g., hype, set/setting), and E. We provide example code in Appendix F illustrating how to implement this mediation strategy. It also shows how our approach accurately recovers the true causal effect. Importantly, the method requires sufficient sample sizes, as theoretical guarantees are asymptotic.

## Sequentially Randomized Experiments

**Figure 5:**
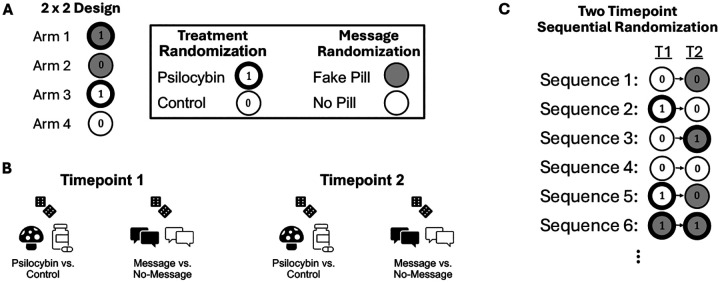
Sequentially Randomized Design. (A) Example 2×2 design at a single timepoint shows that for a single timepoint there are four possible arms/sequences from the treatment × message combination. At each timepoint, we indicate psilocybin with ① and control with ⓪. Similarly, we indicate expectancy manipulation message and no-message with gray and white, respectively. (B) In a two timepoint sequentially randomized experiment, we can separately randomize the treatment (psilocybin/control) and the message at each timepoint. These randomizations can be conducted marginally, or conditional on observed variables such as baseline covariates (e.g., age, sex). At a second timepoint, one could again randomize marginally, or conditional on the response of a participant (e.g., expectancy, depression levels) to previous treatments/messages. This could be used to, for example, increase the chances that psilocybin-treated participants exhibit comparable expectancy levels to control participants. (C) A two timepoint sequentially randomized experiment yields a collection of “arms”, each with different combinations/sequences (often called “regimes” or “policies”) of treatment and messages. “T1” and “T2” indicates timepoints one and two, respectively.

**Figure 6:**
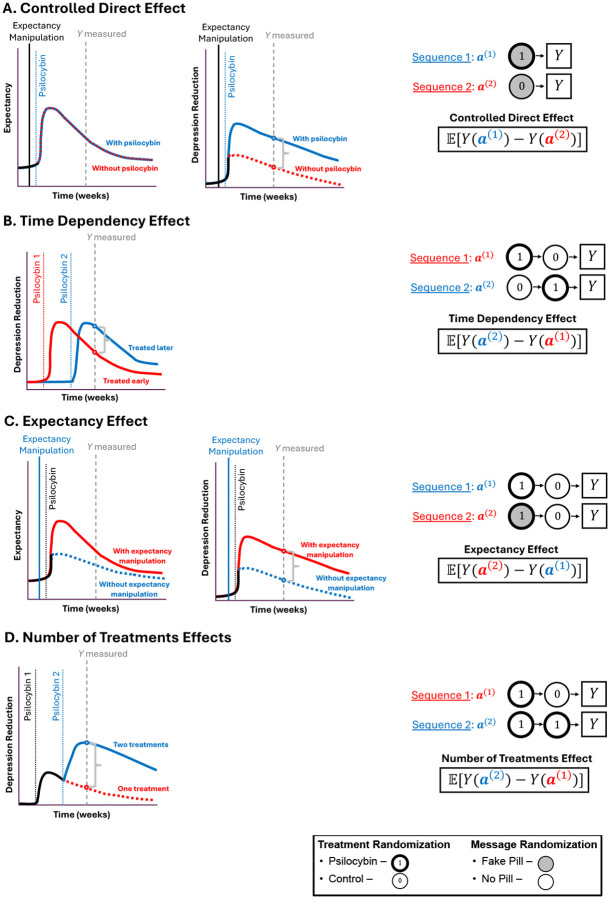
Example causal effects that can be tested in psychedelic RCTs. Each row contains (left) an illustration of potential causal effects on the outcome (depression) and expectancy (where relevant). On the right, we show treatment sequences/combinations (“regimes”/“policies”) of treatment/control and expectancy manipulation messages/no-messages, along side relevant causal effects (“estimands”) that can be estimated and tested with, for example, a Marginal Structural Model. We formalize these causal effects in Appendix E.6. The hypothetical depression and expectancy levels are shown in red/blue lines to differentiate different scenarios (e.g., with vs. without expectancy manipulation). Vertical lines indicate timepoints when expectancy manipulation messages and/or psilocybin treatment are administered, and Y is measured. We use a gray bracket measuring the distance between two dots to show the target causal effect to be estimated by the equation on the right. (A) Controlled Direct Effect in an idealized setting. The figures on the left illustrates the quantity targeted: a difference in depression levels between psilocybin/control arm participants (middle) in a world where the expectancy levels are identical in psilocybin and control arms (left). As indicated by the Treatment Sequences $a^{\left(1\right)},a^{\left(2\right)}$, all participants in the sample are administered an expectancy manipulation message (gray fill), but includes both psilocybin (①) and control arm (⓪) participants. (B) Time Dependency Effects compares the durability of a psilocybin treatment by comparing depression levels between subsets of participants who received treatment at either early or late timepoints. (C) Expectancy Effects captures the causal effect of expectancy manipulation vs. no-manipulation among a subset of participants administered psilocybin. (D) Number of Treatments effects quantifies the causal effect of repeated administrations of psilocybin (one vs. two). This strategy can be used to compare other contrasts (e.g., two vs. zero treatments).

Sequential randomization of both expectancy manipulations and the treatment provides a strategy to probe the effect of treatment sequences that can answer questions related to dose, effect-durability, and expectancy, not possible with single timepoint designs. We illustrate a two timepoint design in Figure 5 for simplicity, although this approach can be applied with more timepoints. This generalizes parallel group dose-response designs. Results from such RCTs can be analyzed with *Marginal Structural Models* (MSMs), arguably the most popular modeling framework for longitudinal causal inference (Hernán et al., 2001; Robins et al., 2000; Robins, 1999) test the effects of specific treatment and message sequences (Robins et al., 2000; Robins, 1999). MSMs can also test the effects of treatment and expectancy sequences by applying the observational causal inference techniques discussed above, thereby echoing and providing a framework for the “call to action […] to thoughtfully integrate longitudinal assessments of expectations into future studies” (Colloca et al., 2023). The RCTs designs allow one to test how treatment and expectancy effects evolve across the study, potentially enabling one to reveal therapeutic benefits obscured by expectancy effects. For example, in Figure 6 we illustrate causal effects related to 1) how long therapeutic benefits of expectancy and/or the psychedelic last, 2) how the outcome changes depending on the number of treatments or expectancy manipulations, 3) the psychedelic’s effects on expectancy, and 4) the CDE. By randomizing assignment at multiple timepoints, participants have multiple opportunities for treatment and are not simply randomized to “one of two arms.” Thus the design may reduce participant pre-occupation with identifying their treatment arm, a common challenge in two-arm RCTs (Szigeti and Heifets, 2024). In Appendix E.6, we propose ways to quantify/test the causal effect of expectancy manipulations and treatment.

## Causal Inference Complements Existing Functional Unmasking Strategies

We believe our modern causal approach can help investigators reason about statistical and design strategies to mitigate bias from unmasking. First, the causal framing highlights the goal of randomization which we believe deserves discussion. For example, Schenberg (2025) argues that RCTs do not deserve the “gold standard status” in part because variable “balance through random allocation […] is not actually granted” in any given sample. Our view is that randomization enables causal inference by ensuring the no unmeasured confounding assumption, not by inducing exact variable balance. While a lack of exact confounder balance between-arms can lead to small sample bias, this does not imply the treatment effect is confounded. Thus, as Schenberg (2025) rightly acknowledges, standard errors should capture uncertainty. Therefore, hypothesis tests of, for example, ATEs remain valid. While successful belief- and/or expectancy-blinding would lead to balance in post-treatment belief and/or expectancy values on average, balance between-arms is not enough to ensure that study results yield causal quantities that correspond to successful masking. This misconception has led to proposed remedies that we believe have drawbacks. For instance, Szigeti and Heifets (2024) propose a resampling strategy to generate adjusted samples with balanced expectancies across treatment arms. This illustrates the need for counterfactual reasoning: confounding occurs at the individual-, not sample-, level and thus reweighting observations to induce sample balance between arms will not resolve the problem. In Appendix E.4.1, we derive the causal quantity that the Szigeti and Heifets (2024) strategy targets and show how it can yield estimates that are so biased that they have the opposite sign of the true direct causal effect.

Second, the DAG suggests a framework for incorporating variables that may strongly influence psychedelic efficacy into analyses, such as, pre-treatment effect modifiers X (e.g., hype, set/setting), or post-treatment expectancy-outcome confounders (e.g., trip experience, post-treatment therapeutic alliance). For example, the FDA recently requested future MDMA applications (U.S. Food and Drug Administration, 2024) “incorporate an assessment” of hype. But how? The DAG illustrates that hype is a potential confounder of the E→Y relationship, and thus should be included in the pre-treatment covariate vector X. However, while hype may modify the A→Y effect, it is *not* a treatment-outcome confounder. Some have suggested running RCTs among low hype participants (Nayak and Zahid, 2025), or using hype as a covariate in a regression model (Aday et al., 2022) in settings prone to functional unmasking. We agree that these strategies will provide valuable insight but we believe that they will not, in general, fully resolve the challenges of unmasking. Such strategies would instead target a *conditional* average treatment effect (CATE): the ATE in a subpopulation that has a specific hype level. Decision-making from RCT results is rarely based on this type of CATE because they may not generalize to other populations. For example, perhaps psychedelics are beneficial among those with positive/neutral pre-treatment attitudes but damaging to those with negative attitudes because they have more bad trips. We discuss this further in Appendix B.

Third, our work clarifies why previous statistical proposals in psychedelic RCTs may not alone resolve challenges from unmasking. For example, Muthukumaraswamy et al. (2021) propose, what we believe, is a *principal stratum* strategy (see expression 14 in their publication). This paper has many virtues, but we believe the principal stratum causal quantity has considerable drawbacks (see, e.g., Stensrud et al. (2023)). Their approach yields estimates that are interpreted as the causal effect of treatment among participants who *would have* the same level of belief in both arms (i.e., the people who *would be* successfully belief-blinded). The downside is that this subpopulation may not exist, and even if it does, it may be highly unrepresentative of the general population. For example, the principal stratum estimate may reflect the psychedelic’s causal effect among people for whom treatment had little therapeutic benefit, thereby explaining why they meet the *would-be successfully belief-blinded* criteria. Moreover, the proposal of Muthukumaraswamy et al. (2021) relies on highly parametric assumptions that may be unrealistic in this setting. We discuss other proposals in Appendix E.3.

Fourth, our approach reveals how RCT design strategies can be improved by explicitly stating causal estimands, corresponding to hypothetical target trials. For example, dose-response psychedelic designs (Goodwin et al., 2022) and/or use of low-dose psychedelics as controls may not alone resolve the expectancy-mediated challenges of functional unmasking because expectancy levels may exhibit a dose-response relationship with the psychedelic dose as we illustrate in Figures 2F–G. That is, the ATE may grow with dose only because expectancy-mediated effects increase with dose. However, testing whether a CDE grows with dose is likely a powerful way to mitigate challenges from unmasking. Similarly, causal theory also explains why “deep blinding” (Matvey et al., 2025; Barrett et al., 2018) may not fully resolve functional unmasking. Deep blinding (Matvey et al., 2025; Barrett et al., 2018) refers to strategies whereby control participants receive an active comparator (e.g., methylphenidate), treatment participants get active treatment (e.g., psilocybin), and all participants are led to believe they could receive any drug from a long list. While deep blinding may lead to belief-blinding, it does not ensure expectancy-blinding (as emphasized in the DMT-salvia example). Thus, while active comparators are currently selected to achieve belief-blinding, the DAG suggests that they should be chosen to induce comparable expectancy levels as the treatment (expectancy-blinding). We formalize this in Appendix E.7. Counterfactual theory also provides technical rationale for deep blinding: it ensures that the *positivity*/overlap causal assumption is met by encouraging sufficient variability in expectancy levels across, for example, treatment arms (discussed in Appendix E). Thus, these designs are remedies for unmasking, when integrated with the formal causal approach.

By pairing an estimand-first strategy with the estimation methods discussed, investigators can rigorously define and estimate causal quantities extending far beyond comparisons of average outcomes across treatment arms. This aligns with the idea of target trials. Indeed, the ICH E9 guideline on statistical principles for RCTs (Kahan et al., 2024) encourages trialists to specify the scientific question in terms of causal estimands and assumptions prior to study design. This contrasts with a more indirect approach that starts with design considerations (e.g., by asking “what is a good control condition for that treatment?” (Colloca and Fava, 2024)). Together, we hope our work provides a basis for strategies that mitigate the unwanted effects of functional unmasking and improve the rigor of RCTs for psychedelic and mental health treatments, more broadly.

## Supplementary Material

1
